# Amputation of the Cervix Uteri

**Published:** 1878-10

**Authors:** 


Amputation of the Cervix Uteri.—This is the subject of an interesting article in the *Annales de Gynecologie*, by Dr. Leblond, with the following conclusions:

1. That the amputation of the cervix ought to be practiced with the uterus in its natural position.

2. That the galvano-caustic method is particularly applicable to uterine cancer, although it may be equally employed in simple hypertrophy.

3. That, in default of galvano-caustic, it is the ecraseur we ought especially to choose in cases of cancer.

4. That the scissors are preferable to the bistoury where one does not care to employ the galvano-caustic or ecraseur.

[The experience of Dr. John Byrne, Brooklyn, who has had greater opportunity for investigation and who has perfected the apparatus of galvano-cautery for uterine purposes to a greater extent perhaps, than any one, abundantly proves that this method is not only preferable for the excision of the cervix in cancer so far as the immediate effects are concerned, but ultimately, as a curative means is its superior.—C. D. P.]—*Lancet and Clinic.*

